# SpyDirect: A Novel Biofunctionalization Method for High Stability and Longevity of Electronic Biosensors

**DOI:** 10.1002/advs.202306716

**Published:** 2023-12-31

**Authors:** Keying Guo, Raik Grünberg, Yuxiang Ren, Tianrui Chang, Shofarul Wustoni, Ondrej Strnad, Anil Koklu, Escarlet Díaz‐Galicia, Jessica Parrado Agudelo, Victor Druet, Tania Cecilia Hidalgo Castillo, Maximilian Moser, David Ohayon, Adel Hama, Ashraf Dada, Iain McCulloch, Ivan Viola, Stefan T. Arold, Sahika Inal

**Affiliations:** ^1^ Computational Bioscience Research Center (CBRC), Biological and Environmental Science and Engineering King Abdullah University of Science and Technology (KAUST) Thuwal 23955‐6900 Saudi Arabia; ^2^ Computer, Electrical and Mathematical Science and Engineering KAUST Thuwal 23955‐6900 Saudi Arabia; ^3^ Department of Chemistry University of Oxford Oxford OX1 3TA UK; ^4^ King Faisal Specialist Hospital & Research Centre (KFSH‐RC) Jeddah 21499 Saudi Arabia; ^5^ Centre de Biologie Structurale (CBS), INSERM, CNRS Université de Montpellier Montpellier F‐34090 France

**Keywords:** cysteine‐peptide linker, nanobody, organic bioelectronics, organic electrochemical transistor, protein sensor, self‐assembled monolayer

## Abstract

Electronic immunosensors are indispensable tools for diagnostics, particularly in scenarios demanding immediate results. Conventionally, these sensors rely on the chemical immobilization of antibodies onto electrodes. However, globular proteins tend to adsorb and unfold on these surfaces. Therefore, self‐assembled monolayers (SAMs) of thiolated alkyl molecules are commonly used for indirect gold–antibody coupling. Here, a limitation associated with SAMs is revealed, wherein they curtail the longevity of protein sensors, particularly when integrated into the state‐of‐the‐art transducer of organic bioelectronics—the organic electrochemical transistor. The SpyDirect method is introduced, generating an ultrahigh‐density array of oriented nanobody receptors stably linked to the gold electrode without any SAMs. It is accomplished by directly coupling cysteine‐terminated and orientation‐optimized spyTag peptides, onto which nanobody‐spyCatcher fusion proteins are autocatalytically attached, yielding a dense and uniform biorecognition layer. The structure‐guided design optimizes the conformation and packing of flexibly tethered nanobodies. This biolayer enhances shelf‐life and reduces background noise in various complex media. SpyDirect functionalization is faster and easier than SAM‐based methods and does not necessitate organic solvents, rendering the sensors eco‐friendly, accessible, and amenable to scalability. SpyDirect represents a broadly applicable biofunctionalization method for enhancing the cost‐effectiveness, sustainability, and longevity of electronic biosensors, all without compromising sensitivity.

## Graphical abstract

SpyDirect creates high‐density, fully oriented nanobody layers on gold gate electrodes of organic electrochemical transistors in a simple water‐based two‐step functionalization process. SpyDirect enhances sensor lifespan and reduces background noise, improving cost‐effectiveness, sustainability, and scalability.

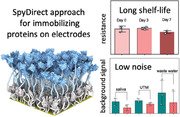

## 1 Introduction

Immunosensors are an important class of affinity‐based biosensing devices that use antibodies or antibody fragments as bioreceptors to detect specific antigen analytes.^[^ ^1^ ^]^ Nanobodies, derived from single‐chain antibodies of camelids, have been particularly successful as bioreceptors in biosensors.^[^ ^2^ ^]^ Nanobodies retain antigen affinity and specificity while offering several advantages, such as being smaller and more soluble while having superior chemical and thermal stability compared to antibodies.^[^ ^3^ ^]^ They are easily and inexpensively produced recombinantly in *Escherichia coli*.^[^ ^4^ ^]^ Their small ≈15 kDa size allows a high packing density when immobilized on a sensor surface, which is critical for rapid and sensitive analyte detection.^[^ ^5^ ^]^

Practical applications of biosensors require receptor immobilization strategies that reduce noise from unspecific binding events and improve sensor robustness and chemical stability. Immobilization strategies for antibodies on sensor electrode surfaces include physical adsorption, matrix entrapment, affinity capture, and covalent chemical coupling.^[^ ^6^ ^]^ Physical adsorption relies on noncovalent intermolecular forces. This method does not control the orientation of antibodies on the sensor surface, resulting in a significant portion of antibodies with concealed binding sites. Adsorption may also lead to partial protein unfolding, reducing the fraction of active receptor units. Moreover, adsorption interactions are relatively weak and sensitive to changes in environmental conditions such as pH and salinity, and receptor units may thus be lost during biosensing or storage. Hence, physical adsorption leads to a patchy and stereochemically heterogeneous biofunctionalization that generates many points of attack by a wide range of chemical or biological aggressors.

Conversely, affinity‐based immobilization permits a stronger noncovalent attachment of antibodies and reduces the risk of concealing or damaging antigen‐binding sites.^[^ ^7^ ^]^ The affinity capture method uses high‐affinity systems such as the avidin–biotin interaction,^[^ ^8^ ^]^ or fusion proteins like A/G which combines two different immunoglobulins G (IgG)‐binding domains.^[^ ^6b^ ^]^ The controlled orientation of protein‐A/IgG antibody surfaces enhances the accessibility of active antigen binding sites, thereby improving sensor performance compared to randomly immobilized antibodies.^[^ ^9^ ^]^ However, even in these affinity capture approaches, the bottom layer of the mediator or capture proteins is still immobilized through random chemical coupling or adsorption. This partial randomness in the biofunctionalization layer and the need for capture proteins increase the complexity and cost of sensor fabrication while compromising sensor robustness. As a result, direct covalent chemical coupling between a modified substrate surface and functional groups on the antibody has become the most commonly employed method in immunosensor design.^[^ ^6b^ ^]^ Typically, the amine (NH_2_) groups of lysine side chains on the antibody are made to react with carboxymethylated dextran layers, glutaraldehyde‐bearing surfaces, or epoxide‐functionalized polymers cast on the sensor surface. In particular, self‐assembled monolayers (SAMs) of thiol‐bearing molecules are widely used as linkers for the covalent immobilization of antibodies (and several other bioreceptors) on surfaces of gold, the workhorse electronic material in the majority of electronic biosensors.^[^ ^10^ ^]^ Chemical coupling through SAMs offers a more direct approach compared to affinity capture and provides greater stability compared to physical adsorption. However, it still presents many of the same drawbacks: the biological receptor is immobilized in a random orientation and location, and both the orientation and chemical treatment can inactivate a portion of the receptor population, exposing and weakening the biofunctionalization surface.

**Figure 1:**
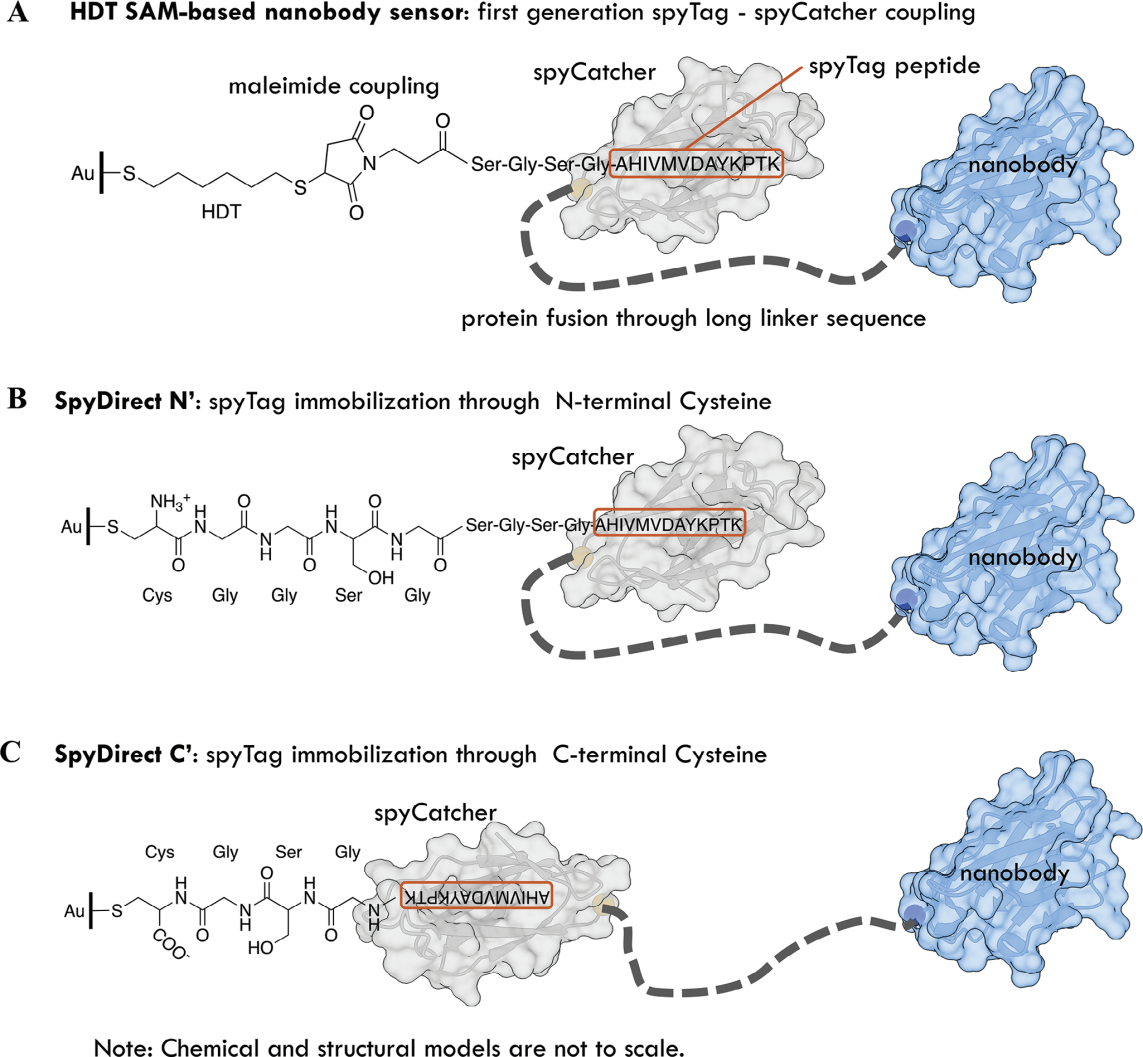
Covalent nanobody conjugation via self‐catalyzing spyTag/spyCatcher bond. A) SAM‐based nanobody immobilization. A synthetic spyTag peptide (marked in a red box) is chemically coupled to the HDT monolayer via maleimide click chemistry. The nanobody‐spyCatcher fusion protein attaches itself to this chemical layer through the autocatalytic formation of a covalent spyTag‐spyCatcher bond. B, C) SpyDirect‐based nanobody immobilization. Direct biofunctionalization of the Au gate electrode with a cysteine‐terminated spyTag‐peptide followed by the coupling of the nanobody‐spyCatcher fusion protein. (B) SpyDirect N′ coupling. The SpyTag is immobilized through an N‐terminal cysteine. (C) SpyDirect C′ coupling. The inverted SpyTag immobilization through a C‐terminal cysteine removes steric constraints for SpyCatcher packing and linker‐nanobody arrangement.

To address these challenges, we recently reported a biofunctionalization strategy for gold electrodes utilized as the gate (and sensing) terminal of an organic electrochemical transistor (OECT). The device involved the covalent conjugation of nanobodies using the spyTag/spyCatcher system on a 1,6 hexanedithiol (HDT) SAM.^[^ ^2a^ ^]^ The spyTag, a 13‐residue peptide, and the spyCatcher, a 113‐residue domain, are derived from a 13 kDa bacterial protein domain.^[^ ^11^ ^]^ When combined in solution, these components spontaneously self‐assemble through the formation of an isopeptide bond. To build electronic sensors, we coupled a maleimide‐activated spyTag peptide to the exposed thiol of the HDT SAM.^[^ ^2a^ ^]^ Subsequently, we introduced nanobody‐spyCatcher fusion proteins, which autonomously assembled into an orientation‐controlled layer on the spyTag‐SAM‐gold surface (**Figure** **1**A). The targeted chemical immobilization of the spyTag peptide (rather than the spyCatcher domain as in a previous protocol^[^ ^11^ ^]^) created an entirely non‐random biofunctionalization architecture and eliminated chemical modifications that may damage the nanobody biorecognition element.^[^ ^12^ ^]^ These advantages became particularly relevant when harnessed in conjunction with the signal amplification capability of the OECT.

OECTs offer unique advantages for the development of immunosensors.^[^ ^13^ ^]^ These devices are well‐suited for integrating bio‐recognition elements on their gate or channel surfaces, enabling the detection of analytes without additional molecules or labels.^[^ ^14^ ^]^ Alongside their high amplification capabilities, OECTs provide the flexibility to adjust the transistor characteristics through modification in channel/gate materials and geometry, creating a customizable amplifier within the same sensing unit. Notably, they can stably operate at low voltages (typically <1 V) in aqueous environments, avoiding undesired redox reactions.^[^ ^13^, ^15^ ^]^ Enhancement‐mode OECTs are particularly suitable for biosensing applications due to their low power consumption, high signal‐to‐noise ratios, and long‐term stability.^[^ ^15^, ^16^ ^]^ Using an enhancement mode OECT gated by gold electrodes functionalized with the layer shown in Figure 1A, we successfully detected target proteins at attomolar‐to‐femtomolar concentrations in less than 15 min.^[^ ^2a^ ^]^ However, the practical application of this sensor, and potentially all similar sensors in the field, is hampered by the lack of durability of the biofunctional layer when exposed to ambient or biological environments. The instability of the alkanethiol SAMs on gold surfaces, the most common bioelectronic interface in sensors, is a key challenge, that is often underestimated. Some reports suggest that SAMs are prone to oxidation shortly after exposure to ambient conditions, often deteriorating within a matter of hours.^[^ ^17^ ^]^ The research groups led by Langer and Agrawal have provided evidence that alkyl‐based SAMs tend to detach from the gold surface when immersed in various biological media, including commonly used phosphate‐buffered saline (PBS) buffer.^[^ ^18^ ^]^ This detachment is primarily attributed to the oxidation of the thiolate headgroup, resulting in the formation of species such as sulfinates and the subsequent desorption of the layer into the surrounding medium. In some cases, the partially decomposed hydrocarbon chains remain at the metal surface.^[^ ^17^ ^]^ Similar instability issues during operation have been documented about thiol‐bearing layers used in aptamer‐based electrodes.^[^ ^19^ ^]^ As a result, the rapid degradation of the very commonly used alkanethiol SAM‐based bioelectronic interface is a limitation that restricts their use to the proof‐of‐concept stage.

In this work, we developed an approach that bypasses chemical SAMs to improve sensor stability and streamline the covalent immobilization of biorecognition units on gold surfaces. In the “SpyDirect” design, we re‐engineered the spyTag peptide so that it can be directly coupled to the gold surface through a C‐terminal cysteine residue, eliminating the need for a SAM layer (Figure 1B,C). The spyCatcher‐nanobody fusion protein then self‐conjugates onto the gold‐spyTag surface. Our SpyDirect immobilization protocol offers several advantages for bioelectronic sensor design. It increases the gold surface coverage with the spyTag peptide while maximizing the packing density of the nanobody, approaching the physical limit. Thanks to these features, compared to the prior SAM‐based OECT sensor design, sensors employing the SpyDirect coupling method exhibit not only superior long‐term stability but also minimal background noise levels when exposed to various biological media, including saliva, universal virus transport medium, and unprocessed wastewater. Moreover, SpyDirect coupling requires just two steps with all components solubilized in water, unlike SAMs that require absolute ethanol, rendering the sensors more cost‐efficient, sustainable, user‐friendly, and conducive to scalability. The SpyDirect coupling approach represents a versatile and universally applicable biofunctionalization strategy that can be seamlessly integrated into the design of any gold‐based bioelectronic system. This innovation promises to advance the field of bioelectronics by enhancing sensor longevity and adaptability to diverse environments, including challenging in vivo settings.

## 2 Results and Discussion

### 2.1 SpyDirect‐Biofunctionalization Strategy

Peptides or proteins can be directly anchored to gold surfaces through the thiol moiety of cysteine residues. However, direct coupling of cysteine‐modified nanobodies was impractical as globular proteins tend to nonspecifically adsorb and unfold on exposed gold surfaces.^[^ ^19^ ^]^ The resulting surface would display a mixture of cysteine‐coupled and randomly adsorbed, folded, and unfolded proteins. These features would strongly depend on the surface properties and stability of each nanobody, hampering sensor repurposing. We, therefore, designed a new spyTag peptide that can be coupled to the gold surface through an N‐terminal cysteine (N‐Cys) (Figure 1B). We separated the cysteine from the spyTag sequence by a flexible 8‐residue spacer to place the spyCatcher domain at a surface distance and orientation comparable to the HDT SAM design (Figure 1A). Molecular modeling showed that this N‐Cys design (like the previous HDT‐based method) projected the spyCatcher linker toward the gold surface rather than away from it. Therefore, we designed an alternative spyTag adapter peptide where the cysteine and a shorter 3 residue spacer were moved to the C‐terminal of the spyTag (C‐Cys; Figure 1C). This design inverts the orientation of the spyTag with respect to the gold surface, leading to a 180° rotation of the spyCatcher domain. This change reduces steric constraints and cluttering within the architecture while maintaining the mobility of the spyCatcher despite reduced peptide length.

### 2.2 Assessing the Efficiency of the SpyDirect Immobilization Protocol

We first evaluated the efficiency of immobilizing nanobody‐spyCatcher using N‐Cys and C‐Cys designs on a gold surface. Using quartz crystal microbalance with dissipation monitoring (QCM‐D), we monitored the change in frequency (Δ*f*) and dissipation (Δ*D*) of a gold crystal during its functionalization with a nanobody targeting green fluorescent protein (GFP). We calculated the cumulative mass gain over time during the two immobilization steps that first attached one of the two different spyTag peptides to the gold surface and then coupled the same nanobody‐spyCatcher fusion protein to the resulting spyTag layer (Figure S1, Supporting Information). In the first step, the shorter (17 amino acids) spyTag peptide with the C‐terminal Cysteine residue (C′ spyDirect) achieved a higher peptide loading density than the longer (22 aa) peptide with the N‐terminal Cysteine (86 × 10^12^ vs 42  × 10^12^ peptides cm^−2^, respectively). The factors influencing the loading of the two peptides likely include molecular size, overall charge distribution, and the vicinity of either a positive (N′‐Cys) or negative charge (C′‐Cys) close to the thiol residue. In the second step, the C′‐Cys spyTag layer captured more nanobody‐spyCatcher fusion protein than the N′‐Cys spyTag layer, (13 × 10^12^ vs 9 × 10^12^ molecules cm^−2^, respectively) (see **Table** **1**). As we illustrate further below, these numbers approach the physical packing limit for the ≈4 nm‐sized spyCatcher and nanobody domains. Unless stated otherwise, we used the SpyDirect C′ configuration (Figure 1C) for further study.

**Table 1:** QCM‐D monitoring of the direct biofunctionalization of GFP nanobody‐spyCatcher using spyTag Cysteine (N‐terminal) or spyTag‐Cysteine (C‐terminal). Three replicates were used.

	SpyDirect N′		SpyDirect C′	
	spyTag	Nanobody‐spyCatcher	spyTag	Nanobody‐spyCatcher
Gained mass [ng cm^−2^]	148 ± 37	526 ± 127	253 ± 11	737 ± 213
Molecule density [cm^−2^]	42 × 10^12^	9 × 10^12^	86 × 10^12^	13 × 10^12^

**Figure 2:**
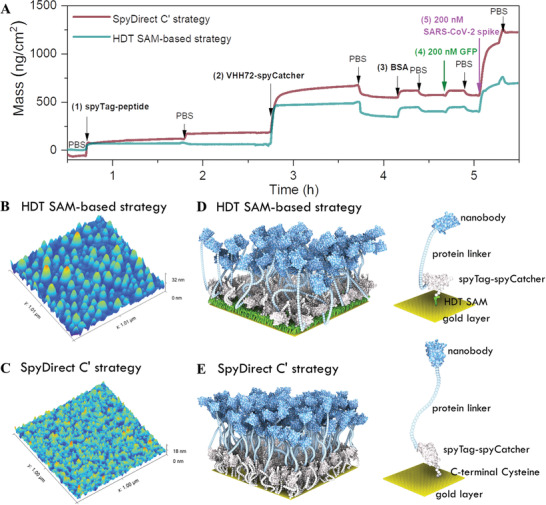
Comparison between the HDT SAM‐based and the SpyDirect‐based nanobody (VHH72) surface. A) QCM‐D profile of the coupling of (1) the spyTag‐peptide (maleimide‐modified peptide versus cysteine‐terminated peptide), (2) VHH72‐spyCatcher fusion protein, (3) bovine serum albumin (BSA), and binding of (4) non‐target GFP and (5) SARS‐CoV‐2 spike protein. HDT SAM‐based nanobody experiment uses an HDT‐coated gold QCM‐D sensor, while SpyDirect uses a bare gold QCM‐D sensor. All the mass was calculated in PBS (10 mm, pH 7.4). Atomic force microscopy (AFM) characterization of B) HDT SAM‐ and C) SpyDirect‐based biofunctional electrode surface in PBS (10 mm, pH 7.4). No BSA was added. A computational rule‐based model generated from the QCM‐D results depicting the D) HDT SAM‐ and E) SpyDirect‐based biofunctional electrode surfaces (to scale).

We next compared the nanobody immobilization between HDT SAM and SpyDirect functionalization methods (**Figure** **2**A). For this assay, we used the VHH72 nanobody recognizing the receptor binding domain (RBD) of the SARS‐CoV‐2 spike protein.^[^ ^20^ ^]^ Changes in Δ*f* and Δ*D* were monitored (see Figure S2 for raw data, Supporting Information), and the cumulative mass gain was calculated as 1) spyTag‐peptide, 2) VHH72‐spyCatcher and 3) bovine serum albumin (BSA; used as a blocking layer to prevent nonspecific adsorption) were introduced to the gold surfaces, followed by washing steps. The gold sensor biofunctionalized through the SpyDirect method accumulated more peptide (241 ± 9 vs 94 ± 32 ng cm^−2^, corresponding to 82 × 10^12^ SpyDirect‐peptide cm^−2^ vs 32 × 10^12^ maleimide–peptide cm^−2^, respectively) and more nanobody (376 ± 18 vs 313 ± 26 ng cm^−2^, corresponding to 7.8 × 10^12^ cm^−2^ vs 6.5 × 10^12^ cm^−2^, respectively). Although the SpyDirect electrode adsorbed less BSA (43 ± 26 vs 90 ± 42 ng cm^−2^), it showed excellent anti‐biofouling performance. When exposed to a nontarget protein (200 nm GFP), the surface gained negligible mass (less than 1 ng cm^−2^). Conversely, the SpyDirect‐based surface bound more than twice as much SARS‐CoV‐2 spike protein than the HDT SAM‐based surface (659 vs 290 ng cm^2^). The spyDirect surface thus captured 4.3 × 10^12^ cm^−2^ target proteins, corresponding to a 55% nanobody occupancy. This result markedly exceeds traditional chemical immobilization schemes. We speculate that this binding capacity is not limited by the availability of binding‐competent nanobodies but by the physical packing of the relatively large spike S1 subunit. The results are summarized in **Table** **2**.

**Table 2:** The amount of peptide, receptor, blocker, as well as target and interferent protein binding on HDT SAM‐ and SpyDirect C′‐functionalized gold surfaces. The averages and the standard deviations are derived from three independent QCM‐D experiments.

	HDT SAM‐based strategy		SpyDirect C′ strategy	
Components introduced in each step	Gained mass [ng cm^−2^]	Molecules [cm^−2]^	Gained mass [ng cm^−2^]	Molecules [cm^−2]^
1) spyTag‐peptide	94 ± 32	3.2 × 10^13^	241 ± 9	8.2 × 10^13^
2) VHH72‐spyCatcher	313 ± 26	6.5 × 10^12^	376 ± 18	7.8 × 10^12^
3) BSA (monomer)	90 ± 42	8.2 × 10^11^	43 ± 26	3.9 × 10^11^
4) non‐target GFP	<4	–	<1	–
5) SARS‐CoV‐2 spike	290	2.3×10^12^	659	5.2 × 10^12^

We used atomic force microscopy (AFM) to investigate the differences between the HDT‐based and SpyDirect‐based VHH72 nanobody surfaces. We monitored the gold electrodes before and after functionalization in a wet state (10 mm PBS, pH 7.4). Compared to bare gold, the nanobody‐modified gold electrode surface had a 1.5 nm increase in its root mean square (RMS) roughness and an 8.4 nm increase in average feature height (Figure S3, Supporting Information). The biofunctionalized surface through SpyDirect coupling had a homogenous distribution of feature sizes while the HDT SAM‐based biofunctional surface showed large aggregates (Figure 2B,C). This difference can be visualized in the relative feature height distribution of these two surfaces (Figure S4, Supporting Information). The SpyDirect‐nanobody surface presents a bell‐shaped curve with a mean value close to 0 nm and a maximum difference in height of less than 5 nm. In contrast, the HDT SAM surface presents an asymmetric distribution of surface features, with sizes up to 15 nm.

To visualize these surfaces, we created a physically simplified rule‐based mesoscale model^[^ ^21^ ^]^ of the layers by combining the available structural information and molecule densities determined by QCM‐D (Figure 2D,E). The model illustrates the tight packing that creates a quasi‐continuous nanobody surface generated through both the HDT SAM‐ or the SpyDirect‐based biofunctionalization methods. However, the re‐orientation of the spyCatcher domain in the C′‐spyDirect design allows for a more relaxed and extended conformation of the spyCatcher‐Nanobody fusion protein. This would offer a ready explanation for the moderately but consistently higher nanobody density and the smoother surface of C′‐spyDirect based biolayers (Tables 1 and 2; Figure S4, Supporting Information).

### 2.3 Chemical and Electrical Characterization of Biofunctionalized Electrodes

**Figure 3:**
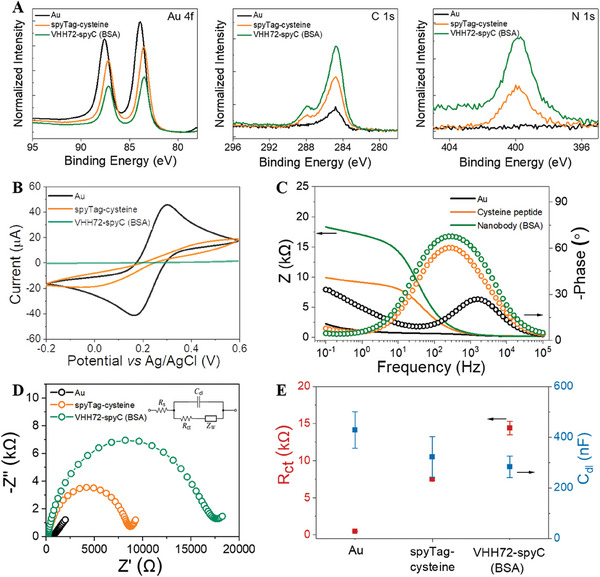
Physiochemical and electrochemical characterization of SpyDirect‐based nanobody functionalized Au electrode. A) High‐resolution XPS spectra for Au 4f, C 1s, and N 1s of the gold surface after immobilization of cysteine‐terminated spyTag‐peptide and the VHH72‐spyCatcher. The VHH72‐spyCatcher buffer contained BSA. B) Cyclic voltammogram, C) Bode plot (solid lines and dotted lines correspond to the magnitude and the phase of the impedance, respectively), D) Nyquist plot of the gold electrode before and after the subsequent functionalization with cysteine‐terminated spyTag peptide, and the VHH72‐spyCatcher. Inset to D is the equivalent circuit model used to fit the impedance spectra. E) The *R*_ct_ and *C*_dl_ of the electrode. The measurements were done in 10 mm [Fe(CN)_6_]^3−/4−^ in 10 mm PBS, pH 7.4.

We used X‐ray photoelectron spectroscopy (XPS) to assess the coverage of the gold electrode surface when functionalized using SpyDirect (**Figure** **3**A). The intensity of the N 1s and C 1s XPS spectra increased with the addition of subsequent layers of SpyDirect peptide and nanobody onto the surface, while the intensity of the Au 4f spectrum decreased, suggesting that the biomolecules cover the gold surface. We used cyclic voltammetry (CV) and electrochemical impedance spectroscopy (EIS) to evaluate the electrical properties of the gold electrode. These characteristics are important for the design of the OECT, as the gate electrode capacitance affects the transistor gain. The bare gold electrode presents the expected reversible peaks for the [Fe(CN)_6_]^3−/4−^ redox couple with a reduction peak current of 42 µA, an oxidation peak current of 44 µA, and a peak‐potential separation of 150 mV (Figure 3B). After adding the peptide layer, the current response decreased, and peaks became further apart from one another, suggesting that the permeability of ions through the peptide layer was drastically reduced. When the nanobody was immobilized, the penetration of the redox probe during the scanning time was further hindered, decreasing the current to almost zero (Figure 3C,D). The impedance spectra were analyzed quantitatively using a Randles equivalent circuit (inset of Figure 3D), which includes the electrolyte resistance (*R*_s_), electric double‐layer capacitance (*C*_dl_) at the electrode/electrolyte interface, charge transfer resistance (*R*_ct_) of the electrode, and Warburg impedance. After the spyTag peptide immobilization on the electrode, the diameter of the semicircle in the high‐frequency region of the Nyquist trace increased significantly, with *R*_ct_ increasing from 0.5 to 7.5 kΩ (Figure 3E). Immobilization of VHH72‐spyCatcher (concurrent with BSA blocking) increased the impedance further, resulting in a large semicircle that spanned the entire frequency range (*R*_ct_ ≈14.4 kΩ). The increase in *R*_ct_ was accompanied by a decrease in *C*_dl_, confirming the high‐density packing of the nanobodies on the electrode.

### 2.4 Detection of SARS‐CoV‐2 Spike Protein with SpyDirect Nanobody‐Based OECTs

OECTs convert small input signals (gate voltage, *V*_G_) into large changes in their output (*I*_D_). The efficiency of this conversion is calculated by taking the first derivative of the transfer curve, defined as transconductance $g_{m}=\frac{\partial I_{D}}{\partial V_{G}}\;$. For the OECT channel, we used a p‐type conjugated polymer p(g0T2‐g6T2) and spin‐cast its solution on the OECT channel (Figure S5, Supporting Information). Without a *V*_G_, the p(g0T2‐g6T2) OECT is in its OFF state (low *I*_D_). When a negative V_G_ is applied, anions from the electrolyte are injected into the film to compensate for the holes, switching the transistor to its ON state (high *I*_D_) (Figure S6a,b, Supporting Information). This OECT has a low power demand (75 µW at sensor operating conditions, Figure S6c, Supporting Information) and high operational stability of the OECT channel (Figures S6d and S7, Supporting Information), demonstrated using a standard Ag/AgCl reference gate electrode.

**Figure 4:**
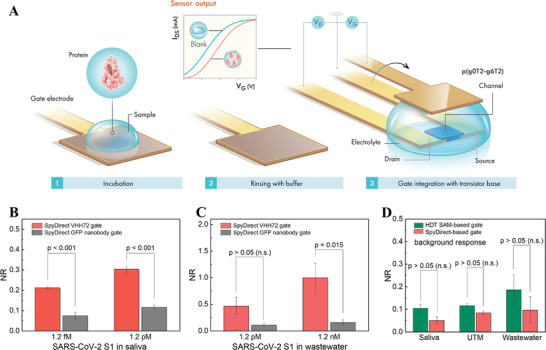
Detection of SARS‐CoV‐2 spike protein in saliva and untreated wastewater. A) Sensor operation. B, C) The response of SpyDirect‐based OECT sensors (nanobody is VHH72) to random concentrations of SARS‐CoV‐2 spike protein in raw saliva and untreated wastewater. D) Background response of SpyDirect‐ and HDT SAM‐based sensors (nanobody is VHH72) to human saliva, UTM, and untreated wastewater. GFP nanobody‐functionalized gate electrodes were used as a control.

Our OECT‐based protein sensor consists of this p‐type channel and the SpyDirect nanobody functionalized gate electrode (**Figure** **4**A). The grounded source contact of the channel and the gate are connected and interface with the electrolyte, which is the measurement solution (phosphate buffer, PB, 40 mm, pH 7.4). We recorded output curves using the functionalized gate electrode washed in buffer solution (i.e., blank) to obtain a baseline signal. The gate electrode was then incubated with 5 µL protein target solution for 10 min and washed thoroughly with binding buffer to remove unbound proteins. The electrode was immersed into the electrolyte on top of the OECT channel to acquire the new output curves. For each case, we determined the normalized response (NR) as the normalized change in OECT *g_m_* calculated at a predetermined drain–source voltage (*V*_D_) and gate voltage (*V*_G_) condition. As a negative control, we used the same channel with a GFP‐nanobody immobilized gate electrode before and after exposure to spike protein, for which it had not shown a significant affinity.^[^ ^2c^ ^]^ If the NR obtained by the VHH72 nanobody gate was higher than the NR with the GFP‐nanobody gate, we concluded that the sample contained the target protein.

To test the sensor under realistic conditions, we prepared different concentrations of the SARS‐CoV‐2 spike protein (S1) subunit in human saliva and wastewater. We recorded transfer curves from multiple SpyDirect gate electrodes functionalized with either VHH72 or GFP nanobody before and after exposure to 5 µL of the target in saliva or untreated wastewater. Figure S8 (Supporting Information) shows transfer curves of devices gated with VHH72 electrodes after their incubation with the target protein in saliva or wastewater. We observed a decrease in current and a shift toward more negative V_G_ values when the VHH72 electrodes were exposed to the protein solution. The devices with the GFP gate showed only negligible changes. Consequently, VHH72 gates responded to the target protein exposure with a larger NR than the GFP nanobody gates (Figure 4B,C), because of the specific interactions between the VHH72 and the spike target protein.^[^ ^2a,c^ ^]^ The binding of the protein to the gate electrode surface decreases its capacitance, which leads to less effective gating, hence, doping of the channel (Figure S9, Supporting Information).

Saliva and wastewater are complex solutions that can cause nonspecific binding to any type of electrode, which is one of the major challenges in the field of biosensors. To assess the extent to which HDT‐based or SpyDirect‐based biofunctionalization allows for unspecific interactions, we calculated the sensor response to these media without target protein using incubation in PB as a “blank” state. SpyDirect OECT sensors had a lower background noise level than the HDT SAM‐based sensors after exposure to raw saliva, universal transport medium (UTM), and untreated wastewater (Figure 4D). These lower background noise levels in complex media suggest that SpyDirect OECT sensors are more reliable under real‐world conditions than HDT SAM counterparts. Unspecific interactions can primarily occur with either exposed gold patches or with hydrophobic/lipophilic surfaces from partially unfolded or aggregated receptor proteins. HDT itself is lipophilic and may, therefore, capture nontarget molecules through hydrophobic interactions. It can, furthermore, create covalent bonds with exposed SH groups and other chemical moieties in the medium. Moreover, the peptide coverage is higher for the SpyDirect surface, which reduces the possibility of gold–protein interaction and, hence, acts as a blocking layer—besides its primary task of linking the nanobody to the surface. Note also that the higher roughness of the HDT‐based nanobody layer may promote nonspecific binding. The reduced background binding may, therefore, be attributed to the absence of HDT and/or the higher density and quality of the SpyDirect nanobody surface.

We evaluated whether the low noise levels of SpyDirect OECT sensors would allow us to identify COVID‐19 infection in human nasopharyngeal swab samples. Nasopharyngeal swabs were collected from hospitalized COVID‐19 patients, confirmed by RT‐PCR at admission (Tabel S1, Supporting Information), and stored in UTM before use. Each sample was tested using two VHH72‐functionalized gates for the actual measurement, an Ag/AgCl reference electrode for channel stability control, and two GFP nanobody‐functionalized gates as a control measurement. All VHH72 gates generated a higher response than the matched GFP nanobody gates, confirming the COVID‐19 positive status of the three samples (Figure S10A, Supporting Information). The OECT channel stability, as evaluated by the Ag/AgCl electrode, was excellent (Figure S10B, Supporting Information). A commercial rapid antigen test kit confirmed the status of samples 2# and 3# but could not detect infection in sample 1#, which had a lower viral load (Figure S10C, Supporting Information). This result suggests that OECT sensors are more sensitive than commercial kits. However, the variation in NR among individual electrodes shows that more effort is needed to improve the reproducibility of gate fabrication and measurement protocol. For now, there is no quantitative correlation between RT‐PCR CT values and OECT NR values, indicating that the sensing technology is only qualitative. This variability also hinders a direct comparison of sensitivity between HDT versus SpyDirect devices.

### 2.5 Stability of SpyDirect Nanobody‐Based Electrodes

**Figure 5:**
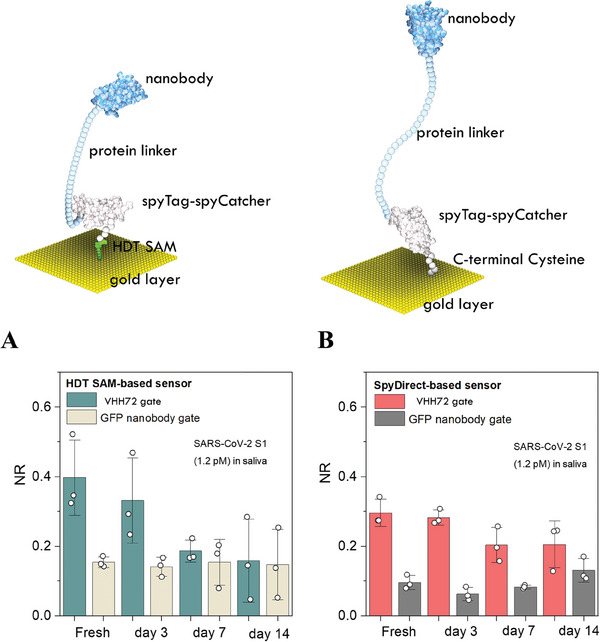
Stability tests of VHH72‐biofunctionalized gates. The normalized response of OECTs to SARS‐CoV‐2 spike protein (S1) (1.2 pm, in saliva) when gated with A) HDT SAM‐based electrodes and B) SpyDirect‐based electrodes. The biofunctionalized gates were stored in 10 mm PBS, pH 74 at 4 °C for 3, 7, and 14 days before use.

Many sensors may perform well soon after they are fabricated. However, commercialization and real‐world applications require high storage stability. Compared to freshly prepared gates, the sensing ability of HDT SAM‐based VHH72 gates decreased continuously over several days of storage in PBS (**Figure** **5**A). After 7 days of storage, the responses of the VHH72 and GFP nanobody gates to SARS‐CoV‐2 S1 were no longer significantly different (Figure 5A). In contrast, SpyDirect gates retained 67% of their initial response after 7 days of storage and the responses of the VHH72 and GFP nanobody gates to 1.2 pm SARS‐CoV‐2 S1 continued to differ significantly (NR = 20 ± 5% and NR < 12 ± 2%, respectively) (Figure 5B).

**Figure 6:**
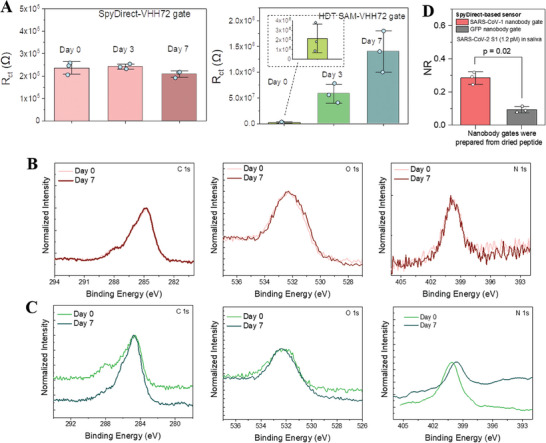
A) The charge transfer resistance (R_ct_) of SpyDirect‐ and HDT SAM‐based gates. The impedance measurements were performed with 10 mm [Fe(CN)_6_]^3−/4−^ in 10 mm PBS, pH 7.4. The Randles circuit model was used to fit the impedance spectra. The biofunctionalized gates were stored in 10 mm PBS, pH 7.4 at 4 °C for 3, 7, and 14 days before use. XPS spectra of C 1s, O 1s, and N 1s of B) SpyDirect‐ and C) HDT SAM‐based gates before and after 7‐day storage in 10 mm PBS, pH 7.4 at 4 °C. D) The response of OECTs gated with SpyDirect‐based electrodes prepared from dried cysteine peptide. The peptide‐modified gates were stored for 7 days in ambient conditions before the nanobody immobilization. All electrode and sensor measurements were done with at least three electrodes.

We investigated the cause of the decline in the performance of HDT‐based sensors using EIS and XPS (**Figure** **6**A). The average R_ct_ value of the SpyDirect nanobody gates was almost the same after 3‐days of storage in PBS (242 vs 237 kΩ) and decreased by 12.5% after 7 days of storage. In contrast, the average R_ct_ values of HDT SAM‐based nanobody gates increased by 26‐fold after 3‐day storage and by 64‐fold after 7‐day storage. Given that the protein has not changed in both strategies, the reason for this instability needs to be sought in the HDT‐SAM layer. Aging‐induced changes were confirmed by chemical shelf‐life stability analysis. C 1s, O 1s, and N 1s XPS spectra of the SpyDirect‐based electrodes showed no change after 7‐day storage in 10 mm PBS, pH 7.4 at 4 °C (Figure 6B), whereas pronounced shifts appeared in the positions, intensities, and widths of peaks recorded for the HDT‐SAM‐based biofunctional layer (Figure 6C). One possible explanation for the shorter shelf life is the high density of exposed thiol moieties, rendering the HDT SAM susceptible to oxidation and possibly cross‐linking.^[^ ^17^, ^18^ ^]^ Increased R_ct_ values argue against widespread peptide/protein detachment. The much higher surface insulation after longer‐term storage hints at the collapse of the biological layer into direct contact with the underlying gold surface. Random (possibly denatured) protein cover would then hinder the diffusion of redox probes toward the substrate.

Finally, one additional advantage of the spyDirect protocol is that the spyTag peptide‐coated surface is not chemically activated, allowing peptide‐coated gate electrodes to be stored dry under ambient conditions before the nanobody immobilization. Indeed, peptide‐coated gates stored for 7 days could be readily functionalized with VHH72 nanobody and effectively detected SARS‐CoV‐2 S1 with a similar NR compared to freshly prepared gates (Figure 6D). This opens up the possibility for the preparation of application‐agnostic peptide‐coated gate electrodes that can be shipped and stored at scale before an application‐specific nanobody receptor protein is introduced. Sensors can then be biofunctionalized on‐site through a simple 15 min incubation with the spyCatcher‐nanobody solution.

## 3 Conclusion

Conventional methods for gold biofunctionalization often rely on thiolated alkyl‐based SAMs. However, the fabrication of these SAMs requires the use of nonaqueous solvents and, at times, deoxygenated environments, rendering the process both environmentally harmful and labor‐intensive. In this study, we introduced a simplified biofunctionalization method, named SpyDirect, suitable for OECT immunosensors gated with Au electrodes and applicable to gold surfaces in general. Instead of using a SAM, SpyDirect employs redesigned cysteine‐terminated spyTag peptides to directly assemble a dense and homogenous nanobody‐spyCatcher fusion protein layer onto the gold gate electrode of the OECT. Compared to our previous spyTag/spyCatcher biofunctionalization based on a thiol SAM, Spydirect demonstrated enhanced sensor shelf‐life and a substantial reduction in non‐specific binding events in challenging media such as saliva and wastewater. The reorientation of the spyCatcher coupling domain increased the density and smoothness of the SpyDirect biolayer compared to the SAM‐based architecture. Our 3D models based on structural and experimental constraints, support the notion of a closely packed nanobody layer with an outward presentation of antigen‐binding surfaces. Consequently, the lower noise and prolonged lifespan of the SpyDirect sensor can likely be attributed to the uniform and patch‐free coverage of the gold electrode, minimizing potential points of attack. Conversely, the poorer chemical stability of the HDT SAM and a less uniform and likely more permeable nanobody layer may account for the higher background noise and faster decline in performance observed in HDT SAM‐based gold electrode‐gated OECTs.

The limited stability of biofunctionalized biosensors is one of the most important roadblocks to their practical applications. SpyDirect biofunctionalization has the potential to emerge as a straightforward, environmentally friendly, and efficacious method for protein biofunctionalization in the next generation of sensing devices, extending beyond immunosensors. The method promises to advance the field of bioelectronics by improving sensor longevity and increasing adaptability to a wide range of environments, possibly including the challenging conditions of implantable devices.

## 4 Experimental Section

### Materials

Sodium chloride, Tween‐20, glycerol, HEPES, bovine serum albumin (BSA), 1,6‐hexanedithiol (HDT), and PBS (pH 7.4) were purchased from Sigma–Aldrich and used as received. All aqueous solutions were prepared with ultrapure water (Millipore Milli‐Q). p(g0T2‐g6T2) was synthesized as reported previously.^[^ ^16^ ^]^ Protein purification materials: Agar, LB broth, 2xYT broth, kanamycin, glucose, isopropyl β‐D‐1‐thiogalactopyranoside (IPTG), BugBuster (Novagen), complete protease inhibitor cocktail (Sigma), benzonase (Novagen), hen egg white lysozyme (Fluka), tris(2‐carboxyethyl)phosphine (TCEP), tris(hydroxymethyl)aminomethane hydrochloride (Tris‐HCl), imidazole, glycerol, dithiothreitol (DTT), ethylenediaminetetraacetic acid (EDTA), D‐desthiobiotin, 10K Amicon ultra spin concentrators (Milipore). Purification columns and materials were purchased from GE. Healthcare: HisTrap‐HP 5 mL, StrepTrap‐HP 5 mL, Superdex75 16/600. Cysteine terminated spyTag peptide and the MCA‐spyTag peptide were synthesized by GenScript Biotech (Singapore). SARS‐CoV‐2 S1 (40591‐V08B1) was purchased from Sino Biological. A universal transport medium kit (UTM, proprietary composition) was obtained from Noble Biosciences, Inc. Untreated wastewater was collected from the KAUST wastewater treatment plant. Saliva was collected from volunteers. Flowflex SARS‐CoV‐2 antigen rapid test kit was bought from a pharmacy. The protocols and procedures involving human saliva were approved by the KAUST Institutional Biosafety and Bioethics Committee (IBEC) (under approval numbers 18IBEC11 and 20IBEC25). All volunteers provided signed consent to participate in the study.

### OECT and Gate Electrode Fabrication

OECTs were fabricated photolithographically using a parylene‐C (PaC) peel‐off method, as reported previously.^[^ ^22^ ^]^ p(g0T2‐g6T2) films and gate electrodes were prepared following the same protocol in a previous report.^[^ ^2a^ ^]^

### Biofunctionalization of the Gate Electrode

HDT SAM‐based sensor surface was prepared following the method reported previously.^[^ ^2a^ ^]^ SpyDirect sensor was prepared as follows. First, 0.1 mg mL^−1^ cysteine terminated peptide with spyTag linker was dissolved in degassed water and applied to the gate electrode for 1 h. The electrodes were rinsed thoroughly with water. Second, 20 µm GFP or VHH72 (with spyCatcher) were dissolved in binding buffer (20 mm HEPES pH 7.4, 150 mm NaCl, 0.02% w/v NaN_3_, 0.05% v/v Tween‐20, 0.1% w/v BSA), and incubated with the peptide‐linked electrodes for 1 h. Subsequently, the nanobody functionalized gate electrodes were rinsed with a binding buffer.

### Electrochemical Measurements

All electrochemical measurements were performed in a conventional three‐electrode setup using a potentiostat (Autolab PGstat128N, MetroOhm). A platinum wire and Ag/AgCl electrodes were employed as the counter and the reference electrodes, respectively. The gold electrode was used as the working electrode. Measurements were carried out in 5 mL of 10 mm PBS solution (pH 7.4) containing 10 mm [Fe(CN)_6_]^3−/4−^. For CV measurements, the potential window of gold was determined typically between −0.2 and 0.6 V, and the scan rate was 100 mV s^−1^. Impedance spectra were recorded at a DC voltage of 0 V versus open circuit potential and an AC modulation of 10 mV over a frequency range of 0.1–100 000 Hz.

### XPS Characterization

XPS measurements were performed using AMICUS/ESCA (1468.6 eV). The source was operated at 10 kV with 10 mA current generating a power of 100 W. The vacuum level of the analysis chamber was maintained at 10^−7^ Pa during the measurements. The obtained spectra were calibrated to reference C 1s at 284.8 eV. The background of XPS spectra was carried out by the Tougaard method.

### QCM‐D Monitoring

QCM‐D measurements were conducted using a Q‐sense analyzer (QE401, Biolin Scientific) following either HDT SAM‐ or SpyDirect‐ biofunctionalization. The piezoelectrically active gold sensors (0.7854 cm^2^) were used. All solutions were injected into the chamber with a flow rate of 100 µL min^−1^, controlled by a peristaltic pump. After ensuring that the sensor was fully covered with the solution, the pump was stopped for static incubation for a certain period of time. All QCM‐D data presented in this work were recorded at the seventh overtone and analyzed using the same method detailed in the previous work.^[^ ^2a^ ^]^ The number of immobilized macromolecules was calculated using the following molecular masses: maleimide‐peptide, 1.78 kDA; SpyDirect‐peptide, 1.78 kDA; nanobody‐spyCatcher protein, 28.39 kDa; VHH72‐spyCatcher protein, 29.05 kDa. The S1 subunit of the SARS‐CoV‐2 spike protein (Sino Biological, #40591‐V08B1) has a protein‐only molecular weight of 76.45 kDa, to which glycosylation adds 16 kDa.^[^ ^23^ ^]^

### AFM

AFM scans were obtained with a Veeco Dimension 3100 Scanning Probe System. In electrolyte topographic scans were conducted using the Bruker SCANASYST‐FLUID module mounted with Scanasyst‐fluid probes commercialized by Bruker (nominal resonant frequency: 150 kHz, spring constant: 0.7 N m^−1^). The sample and probe were both immersed in 10 mm PBS, pH 7.4 at room temperature while scanning. Gwyddion software was used for statistical data and post‐treatment.

### Proteins

Design of nanobody‐spyCatcher fusion proteins, and preparation of SARS‐CoV‐2 S1 were done following the protocol as published previously.^[^ ^2a^ ^]^ Lab‐produced proteins were desalted into DTT‐free storage buffer (20 mm HEPES pH 7.4, 150 mm NaCl, 0.05% v/v Tween‐20, 0.02% w/v NaN_3_) before use. Protein concentrations were assessed spectrophotometrically (Nanodrop, Thermofisher). Protein dilutions were prepared in standard sensor binding buffer (20 mm HEPES pH 7.4, 150 mm NaCl, 0.05% v/v Tween‐20, 0.02% w/v NaN_3_, 0.1% w/v BSA) or wastewater. For the measurement of saliva samples, a complete protease inhibitor cocktail with EDTA (Roche) was added at four times the concentration recommended by the manufacturer, and 0.5% w/v BSA was included. The saliva used for original sensor characterization with recombinant proteins was freshly self‐collected from healthy volunteers and used on the same day. The wastewater was confirmed COVID‐19‐negative by RT‐qPCR before use.

### Human Patient Sample Preparation and Testing

The nasopharyngeal swabs used in this study were collected from human subjects as part of registered protocols approved by the Institutional Review Board of the King Faisal Specialist Hospital and Research Center (KFSH‐RC) and KAUST Institutional Biosafety and Bioethics Committee (IBEC) (under approval numbers 18IBEC11 and 20IBEC25), and the National Committee of BioEthics, Saudi Arabia (registration number HAP‐02‐J‐042). All volunteers provided signed consent to participate in the study. Three nasopharyngeal swabs collected from outpatients with COVID‐19 were stored in UTM at −20 °C and tested with PCR. Raw samples were measured after 1:3 dilution in a virus‐inactivating lysis buffer (50 mm Tris (pH 7.4), 250 mm NaCl, 1% Nonidet P‐40, 0.02% NaN_3_, 0.5% BSA and 4x cOmplete) by SARS‐CoV‐2 antigen rapid test kit and OECT sensor, respectively.

### OECT Sensor Characterization and Operation

Electrical characterization of the transistor was carried out with a Keithley source meter, which was used to apply the drain and gate voltages. All the measurements were conducted under ambient conditions. A PDMS well was glued on the channels and filled with 200 µL of 40 mm phosphate buffer (PB), pH 7.4, as an electrolyte. The OECT channel was stabilized with reference Ag/AgCl gate by repeating output curves (I*_D_*–V*_D_*). The steady‐state measurements of the OECTs were performed by acquiring drain current (*I_D_*) versus drain voltage (*V_D_*) at gate voltages (*V_G_*) varying between 0.2 and −0.4 V (step 0.05 V). *V_D_* was swept from 0 to −0.4 V. For each measurement (before or after analyte incubation), three *I_D_*–*V_G_* curves were plotted from the output characteristics, and the third *I_D_*–*V_G_* was used to calculate the g_m_ as the curves stabilized during this scan. All the nanobody gates were kept in PB (40 mm, pH 7.4) for at least 10 min before sensing. A baseline in PB as blank was obtained before sensing, and the read‐out signals obtained were used as a baseline (*g*_*m*0_). The nanobody functionalized gate electrode was incubated at room temperature for 10 min (pipetting 30 s every 3 min) with 5 µL sample solution. The sensor performance is defined by the normalized response (NR) calculated from the change in g_m_ after analyte binding, normalized by *g*_*m*0_. The NR was used to determine a calibration curve according to the following equation:

(1) $$NR=|\left(g_{mD}-g_{m0}\right)|/g_{m0}$$

## Conflict of Interest

A US provisional application with No. 63/280,887 related to this work was filed by S.I., S.A., R.G., K.G., S.W., and A.K. in 2021.

## Author Contributions

K.G. and R.G. contributed equally to this work. S.I., S.A., and R.G. conceived the research, designed the experiments, and supervised the work. K.G., J.P.A., and T.C.C.H. fabricated the OECT devices. K.G. and T.C. functionalized the gate electrodes, conducted EIS, and QCM‐D experiments, and performed the sensing experiments. K. G. and D.O. analyzed the QCM‐D data. K.G., S.W., A.K., and T. C. performed the clinical sample testing. A.K. conducted the stability testing of OECT in PBS electrolyte. S.W. performed XPS measurements, and V.D. conducted AFM experiments. R.G., Y. R., and E.D.G. designed and produced recombinant proteins. O.S. and I.V. visualized the nanobody functionalized gold electrodes. A. D. organized the clinical samples. A.H. developed the LabView codes to operate the OECTs. M. M. and I. M. provided the p‐type OECT material. K.G. drafted the manuscript and coordinated the experiments. All the authors contributed to scientific discussions and manuscript writing.

## Supporting information

Supporting Information

## Data Availability

The data that support the findings of this study are available from the corresponding author upon reasonable request.

## References

[1] a) A. P. F. Turner , Science 2000, 290, 1315; 11185408 10.1126/science.290.5495.1315

[2] a) K. Guo , S. Wustoni , A. Koklu , E. Díaz‐Galicia , M. Moser , A. Hama , A. A. Alqahtani , A. N. Ahmad , F. S. Alhamlan , M. Shuaib , A. Pain , I. Mcculloch , S. T. Arold , R. Grünberg , S. Inal , Nat. Biomed. Eng. 2021, 5, 666; 34031558 10.1038/s41551-021-00734-9

[3] S. Steeland , R. E. Vandenbroucke , C. Libert , Drug Discovery Today 2016, 21, 1076. 27080147 10.1016/j.drudis.2016.04.003

[4] a) T. De Meyer , S. Muyldermans , A. Depicker , Trends Biotechnol. 2014, 32, 263; 24698358 10.1016/j.tibtech.2014.03.001

[5] D. Saerens , F. Frederix , G. Reekmans , K. Conrath , K. Jans , L. Brys , L. Huang , E. Bosmans , G. Maes , G. Borghs , S. Muyldermans , Anal. Chem. 2005, 77, 7547. 16316161 10.1021/ac051092j

[6] a) F. Rusmini , Z. Zhong , J. Feijen , Biomacromolecules 2007, 8, 1775; 17444679 10.1021/bm061197b

[7] Y. Liu , J. Yu , Microchim. Acta 2016, 183, 1.

[8] H. C. Yoon , M.‐Y. Hong , H.‐S. Kim , Anal. Biochem. 2000, 282, 121. 10860508 10.1006/abio.2000.4608

[9] a) P. Peluso , D. S. Wilson , D. Do , H. Tran , M. Venkatasubbaiah , D. Quincy , B. Heidecker , K. Poindexter , N. Tolani , M. Phelan , K. Witte , L. S. Jung , P. Wagner , S. Nock , Anal. Biochem. 2003, 312, 113; 12531195 10.1016/s0003-2697(02)00442-6

[10] a) S. Flink , F. C. J. M. V. Veggel , D. N. Reinhoudt , Adv. Mater. 2000, 12, 1315;

[11] B. Zakeri , J. O. Fierer , E. Celik , E. C. Chittock , U. Schwarz‐Linek , V. T. Moy , M. Howarth , Proc. Natl. Acad. Sci. USA 2012, 109, E690. 22366317 10.1073/pnas.1115485109 PMC3311370

[12] W. Yang , J. Justin Gooding , D. Brynn Hibbert , J. Electroanal. Chem. 2001, 516, 10.

[13] a) D.‐J. Kim , N.‐E. Lee , J.‐S. Park , I.‐J. Park , J.‐G. Kim , H. J. Cho , Biosens. Bioelectron. 2010, 25, 2477; 20435461 10.1016/j.bios.2010.04.013

[14] a) H. Liu , A. Yang , J. Song , N. Wang , P. Lam , Y. Li , H. K.‐W. Law , F. Yan , Sci. Adv. 2021, 7, eabg8387; 34524851 10.1126/sciadv.abg8387 PMC8443172

[15] a) D. Khodagholy , J. Rivnay , M. Sessolo , M. Gurfinkel , P. Leleux , L. H. Jimison , E. Stavrinidou , T. Herve , S. Sanaur , R. M. Owens , Nat. Commun. 2013, 4, 1; 10.1038/ncomms3133 PMC3717497 23851620

[16] M. Moser , T. C. Hidalgo , J. Surgailis , J. Gladisch , S. Ghosh , R. Sheelamanthula , Q. Thiburce , A. Giovannitti , A. Salleo , N. Gasparini , A. Wadsworth , I. Zozoulenko , M. Berggren , E. Stavrinidou , S. Inal , I. Mcculloch , Adv. Mater. 2020, 32, 2002748. 10.1002/adma.202002748 32754923

[17] a) M. H. Schoenfisch , J. E. Pemberton , J. Am. Chem. Soc. 1998, 120, 4502;

[18] a) N. T. Flynn , T. N. T. Tran , M. J. Cima , R. Langer , Langmuir 2003, 19, 10909;

[19] A. Shaver , S. D. Curtis , N. Arroyo‐Currás , ACS Appl. Mater. Interfaces 2020, 12, 11214. 32040915 10.1021/acsami.9b22385

[20] D. Wrapp , D. De Vlieger , K. S. Corbett , G. M. Torres , N. Wang , W. Van Breedam , K. Roose , L. Van Schie , M. Hoffmann , S. Pöhlmann , B. S. Graham , N. Callewaert , B. Schepens , X. Saelens , J. S. Mclellan , Cell 2020, 181, 1004. 32375025 10.1016/j.cell.2020.04.031 PMC7199733

[21] N. Nguyen , O. Strnad , T. Klein , D. Luo , R. Alharbi , P. Wonka , M. Maritan , P. Mindek , L. Autin , D. S. Goodsell , I. Viola , IEEE Trans Vis Comput Graph 2021, 27, 722. 33055034 10.1109/TVCG.2020.3030415 PMC8642830

[22] D. Ohayon , G. Nikiforidis , A. Savva , A. Giugni , S. Wustoni , T. Palanisamy , X. Chen , I. P. Maria , E. Di Fabrizio , P. M. F. J. Costa , I. McCulloch , S. Inal , Nat. Mater. 2020, 19, 456. 31844278 10.1038/s41563-019-0556-4

[23] L. van Oosten , J. Altenburg Jort , C. Fougeroux , C. Geertsema , F. van den End , A. C. Evers Wendy , H. Westphal Adrie , S. Lindhoud , W. van den Berg , C. Swarts Daan , L. Deurhof , A. Suhrbier , T. Le Thuy , S. Torres Morales , K. Myeni Sebenzile , M. Kikkert , F. Sander Adam , A. de Jongh Willem , R. Dagil , A. Nielsen Morten , A. Salanti , M. Søgaard , M. P. Keijzer Timo , D. Weijers , H. M. Eppink Michel , H. Wijffels René , M. van Oers Monique , E. Martens Dirk , P. Pijlman Gorben , mBio 2021, 12, e01813. 34634927 10.1128/mBio.01813-21 PMC8510518

