# Supplementary material for: SpyDirect: A Novel Biofunctionalization Method for High Stability and Longevity of Electronic Biosensors
Source: Adv Sci (Weinh). 2023 Dec 31;11(27):2306716. doi: 10.1002/advs.202306716 (PMC11251562; doi:10.1002/advs.202306716)
Supplement: Supplementary file 1 — Supporting Information [file ADVS-11-2306716-s001.pdf]

ADVANCED SCIENCE
Open Access

Supporting Information

for *Adv. Sci.*, DOI 10.1002/advs.202306716

SpyDirect: A Novel Biofunctionalization Method for High Stability and Longevity of Electronic Biosensors

Keying Guo, Raik Grünberg, Yuxiang Ren, Tianrui Chang, Shofarul Wustoni, Ondrej Strnad, Anil Koklu, Escarlet Díaz-Galicia, Jessica Parrado Agudelo, Victor Druet, Tania Cecilia Hidalgo Castillo, Maximilian Moser, David Ohayon, Adel Hama, Ashraf Dada, Iain McCulloch, Ivan Viola, Stefan T. Arold and Sahika Inal**

Supplementary information for

SpyDirect: A Novel Biofunctionalization Method for High Stability and Longevity of Electronic Biosensors

Keying Guo,[1] † Raik Grünberg,[1] † Yuxiang Ren,[1] Tianrui Chang,[1] Shofarul Wustoni,[1] Ondrej Strnad,[2] Anil Koklu,[1] Escarlet Díaz-Galicia,[1] Jessica Parrado Agudelo,[1] Victor Druet,[1] Tania Cecilia Hidalgo Castillo,[1] Maximilian Moser,[3] David Ohayon,[1] Adel Hama,[1] Ashraf Dada,[4] Iain McCulloch,[3] Ivan Viola,[2] Stefan T. Arold,[1, 5] Sahika Inal[1]**

1. King Abdullah University of Science and Technology (KAUST), Computational Bioscience Research Center (CBRC), Biological and Environmental Science and Engineering, Thuwal, 23955-6900, Saudi Arabia
2. KAUST, Computer, Electrical and Mathematical Science and Engineering, Thuwal, 23955-6900, Saudi Arabia
3. University of Oxford, Department of Chemistry, Oxford, OX1 3TA, United Kingdom
4. King Faisal Specialist Hospital & Research Centre (KFSH-RC), Jeddah, 21499, Saudi Arabia
5. Université de Montpellier, Centre de Biologie Structurale (CBS), INSERM, CNRS, Montpellier, F-34090, France.

*Corresponding authors Email: stefan.arold@kaust.edu.sa; sahika.inal@kaust.edu.sa

†These authors contributed equally to this work.

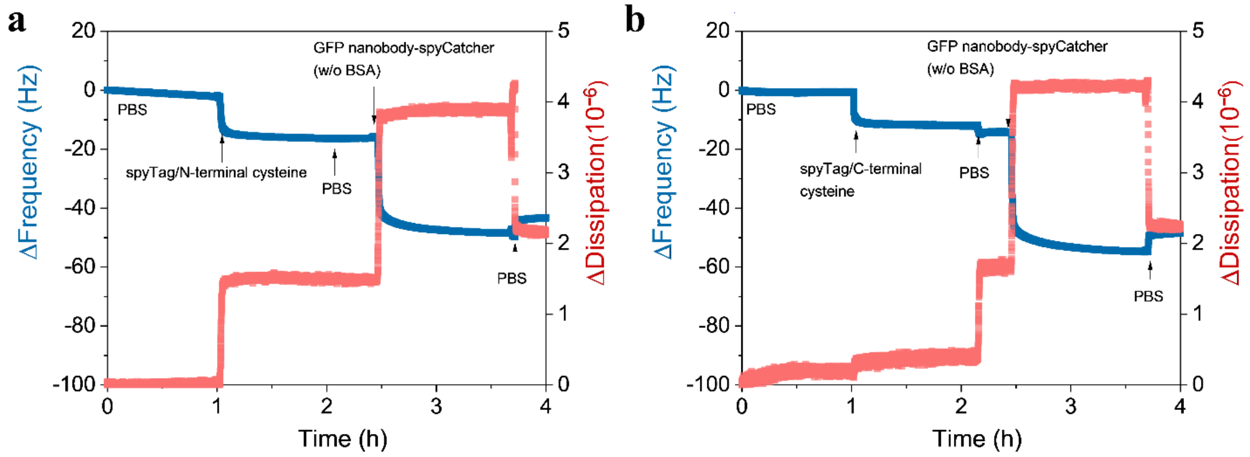

Figure S1. QCM-D monitoring of the GFP nanobody immobilization through coupling with (a) spyDirect N' or (b) spyDirect C'. The QCM-D signals including the change in frequency (Δf)

and dissipation (ΔD) are plotted over time as (1) spyTag-cysteine, (2) GFP nanobody-spyCatcher was introduced to the system, followed by washing steps.

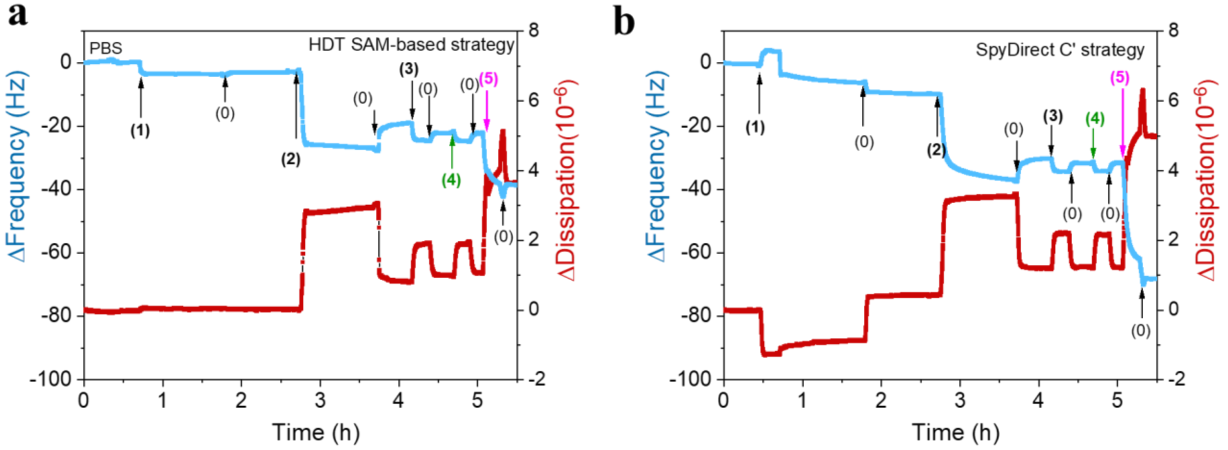

Figure S2. Monitoring VHH72 nanobody/spyCatcher immobilization and protein binding through (a) HDT SAM-based and (b) SpyDirect C' strategy using OCM-D. HDT SAM-based nanobody started from an HDT-coated gold QCM-D sensor, while SpyDirect-nanobody started from a bare gold QCM-D sensor. The following injections were done: (1) the spyTag-peptide (maleimide-modified peptide vs. cysteine-terminated peptide), (2) VHH72-spyCatcher fusion protein, (3) BSA, (4) non-target GFP and (5) SARS-CoV-2 spike protein followed by washing steps (0)

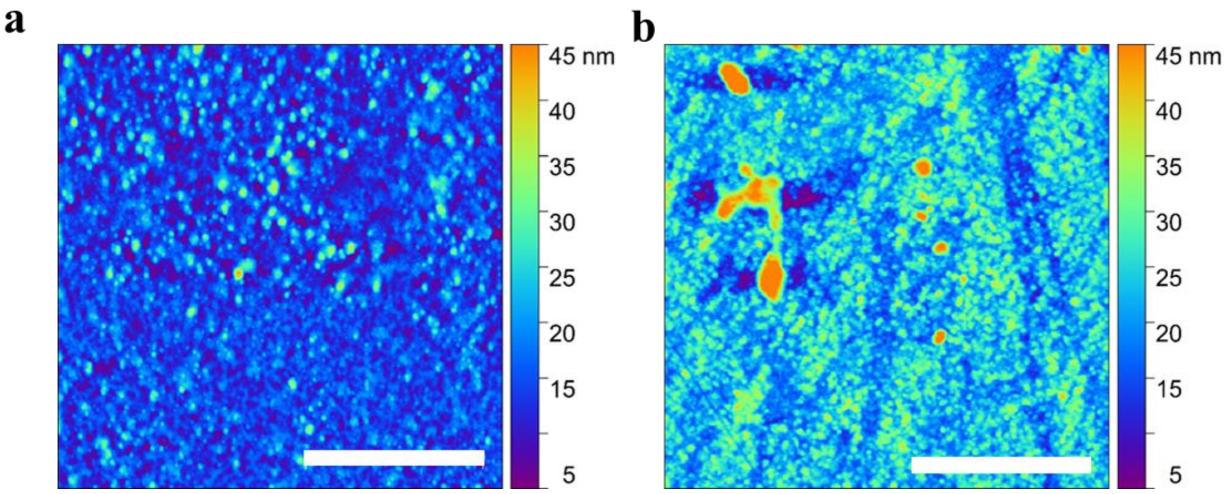

Figure S3. Atomic force microscopy (AFM) characterization of the biofunctionalized gate electrode with nanobody. AFM topography image of **(a)** a bare gold electrode, **(b)** the same electrode after immobilization of SpyCatcher-nanobody. The scanned area is 5 μm × 5 μm, scale bar is 2 μm.

Atomic force microscopy (AFM) was used to confirm the change in the surface roughness and feature height during biofunctionalization. Before immobilizing any biomolecules, the root mean square (RMS) roughness of the electrode is 4.5 nm, and the mean height of the grains is 14.7 nm. After incubating with the nanobody solution, large particles were observed on the gate electrode surface. The RMS roughness of the nanobody-modified electrodes increased to 6.0 nm. The mean feature height increased by 8.4 nm (from 14.7 to 23.1 nm), verifying the successful immobilization of nanobody and BSA.

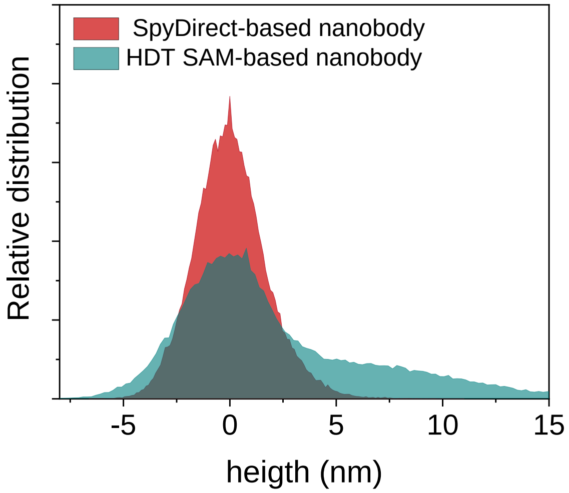

Figure S4. Relative distribution of feature heights of nanobody functionalized surfaces prepared through the two methods (HDT SAM vs. SpyDirect) determined using Gaussian fitting.

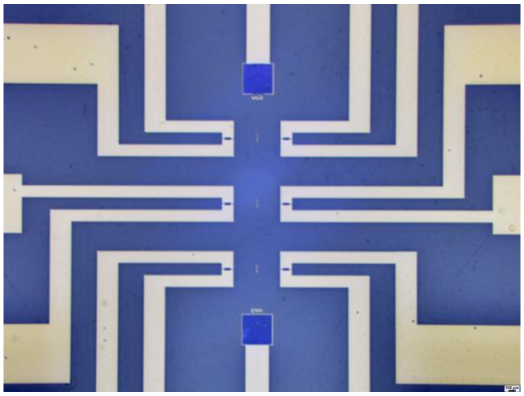

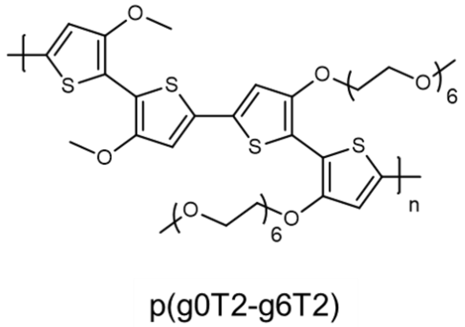

Figure S5. Optical picture of one chip comprising 6 channels (100 µm × 10 µm) covered with p-type material p(g0T2-g6T2) film prepared by spin-coating. The polymer-coated microelectrodes shown in the image were used for the electrochemical characterization of the material.

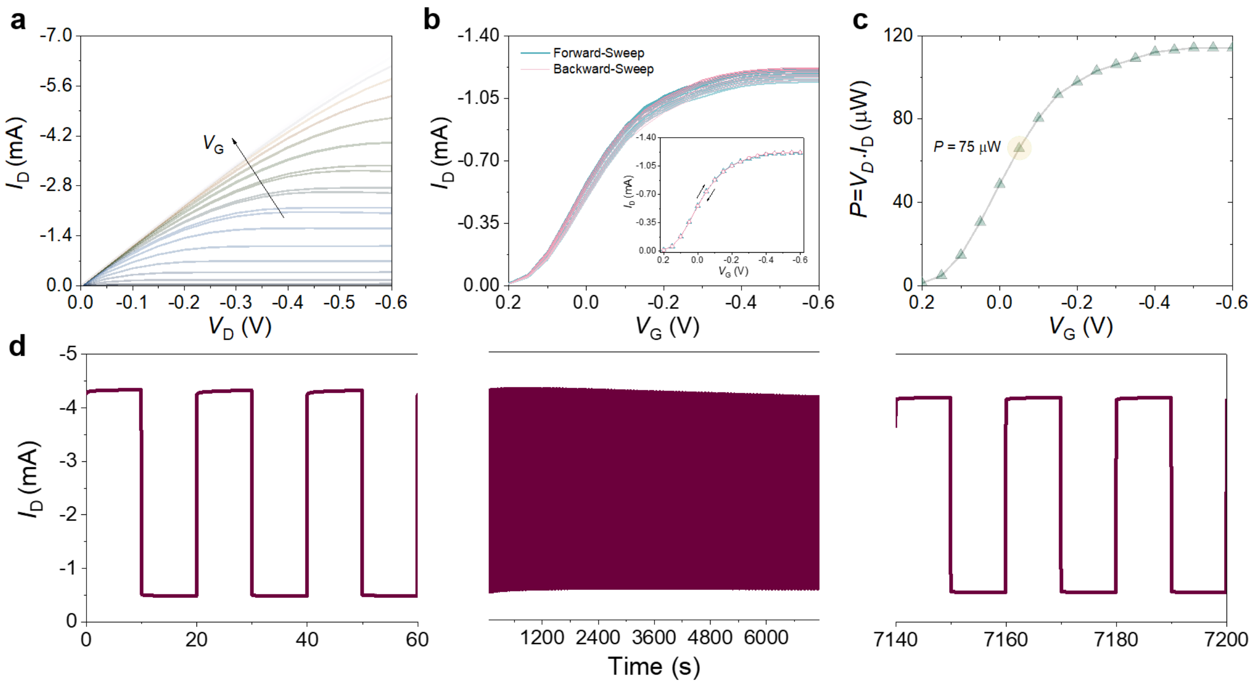

Figure S6. **(a)** The output characteristics of p(g0T2-g6T2) devices with V_G varying from 0.2 V to -0.6 V. The arrows indicate the scan direction of V_G. The scan rate was 50 mV/s. **(b)** Ten repetitive transfer curves of exemplary devices recorded at V_D = -0.1 V. The arrows in the inset figure indicate the scan direction hysteresis for the last repetition. **(c)** The calculated power consumption at different V_G (V_D = -0.1 V). **(d)** Transient characteristics of z p(g0T2-g6T2) device over an hour of continuous ON and OFF biasing (10 seconds each) at the gate electrode. The operation conditions are V_D = -0.5 V, V_G = -0.5 V (or 0 V). All measurements were performed in 10 mM PBS using an Ag/AgCl as the gate electrode.

Figure S6a shows that at low V_D, the increase in I_D is significant, followed by a saturation regime at higher V_D, consistent with accumulation mode OECT operation. The device showed minimal hysteresis with almost identical behavior, as observed from forward and backward voltage scans (**Figure S6b**). The p(g0T2-g6T2) transistors had low OFF-currents on the order of 10 μA, and an ON/OFF ratio of up to 100 at V_G, which led to maximum g_m in the saturation regime (**Figure S6b**). Our OECT had a lower power demand (75 μW at V_G = -0.05 V, V_D = -0.1 V) when operated at the subthreshold regime, which yielded the maximum sensor NR values (**Figure S6c**). We investigated the operational stability of our devices by switching them "ON" and "OFF" for 10 s (V_G = - 0.5V or 0 V) each at constant V_D= -0.5 V and recording the I_D over 360 cycles performed within 2 hours (**Figure S6d**). The device retained 98% of its initial current after cycling. Small gate voltages applied to keep the device in its ON-state reduce the risk of material instability for long-term use requirements.

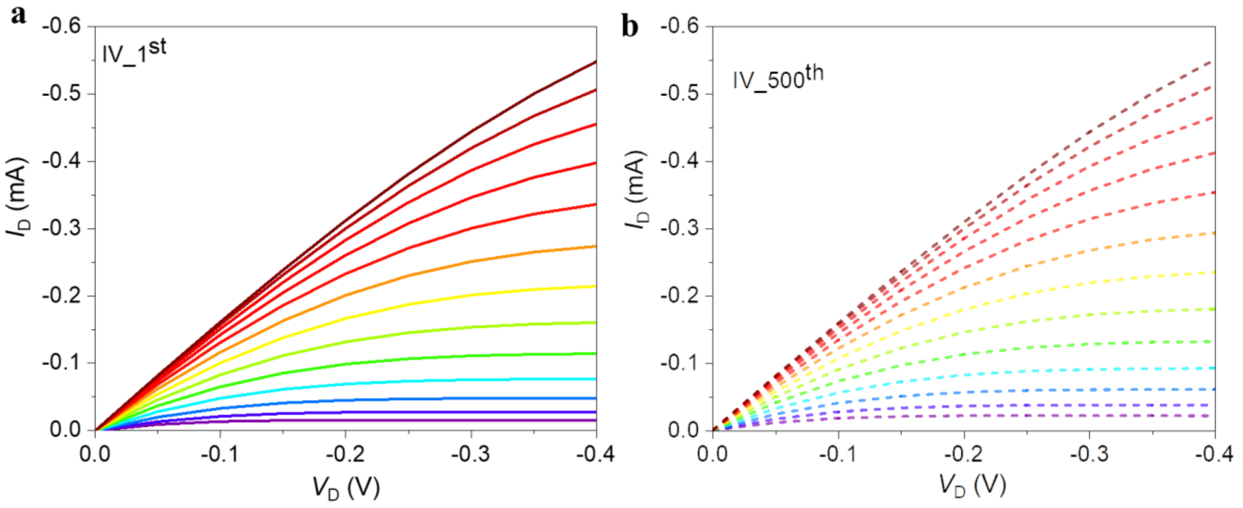

Figure S7. The output characteristics obtained by sweeping V_D from 0 to -0.4 V and V_G from 0.2 V to -0.4 V in PB (40 mM, pH 7.4). **(a)** The first I-V and **(b)** the 500th I-V curves. The change of channel current at V_D = V_G = -0.4 V is 0.8%. All measurements were performed in PB (40 mM, pH 7.4) using an Ag/AgCl as the gate electrode.

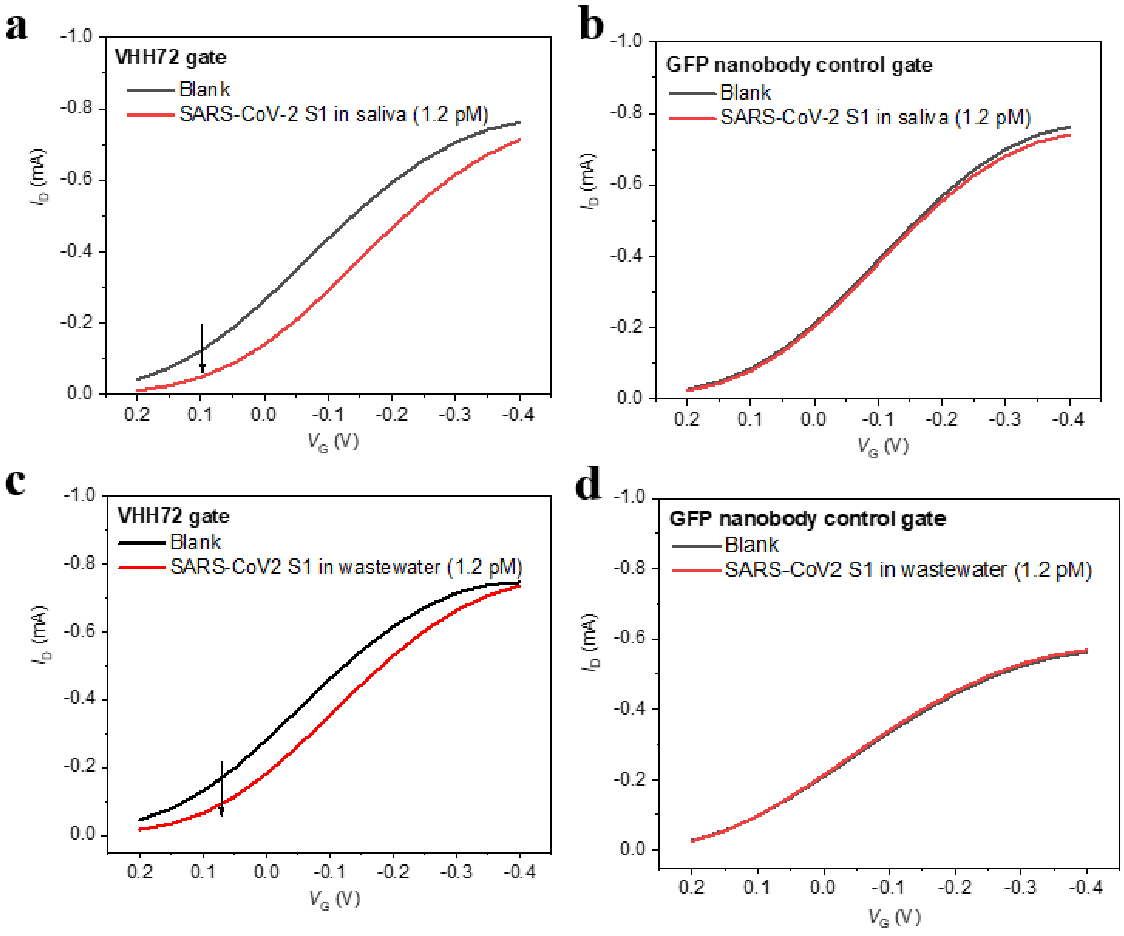

Fig. S8. Typical transfer (I_D vs. V_G) characteristics of the p(g0T2-g6T2) OECT gated with the (a, c) VHH72 nanobody or (b, d) GFP nanobody immobilized gold electrode before (blank) and after its incubation with SARS-CoV-2 S1 spiked either in saliva or in wastewater.

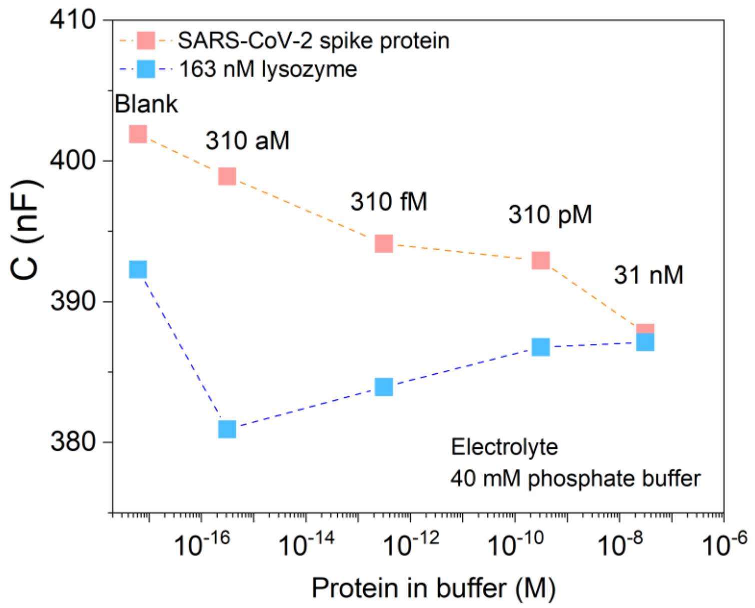

Figure S9. The electrochemical capacitance of a VHH72 electrode upon protein binding. The EIS measurements were performed in 40 mM PB, pH 7.4. The data were fitted using Randles circuit to extract the capacitance. Lysozyme was used as a negative control.

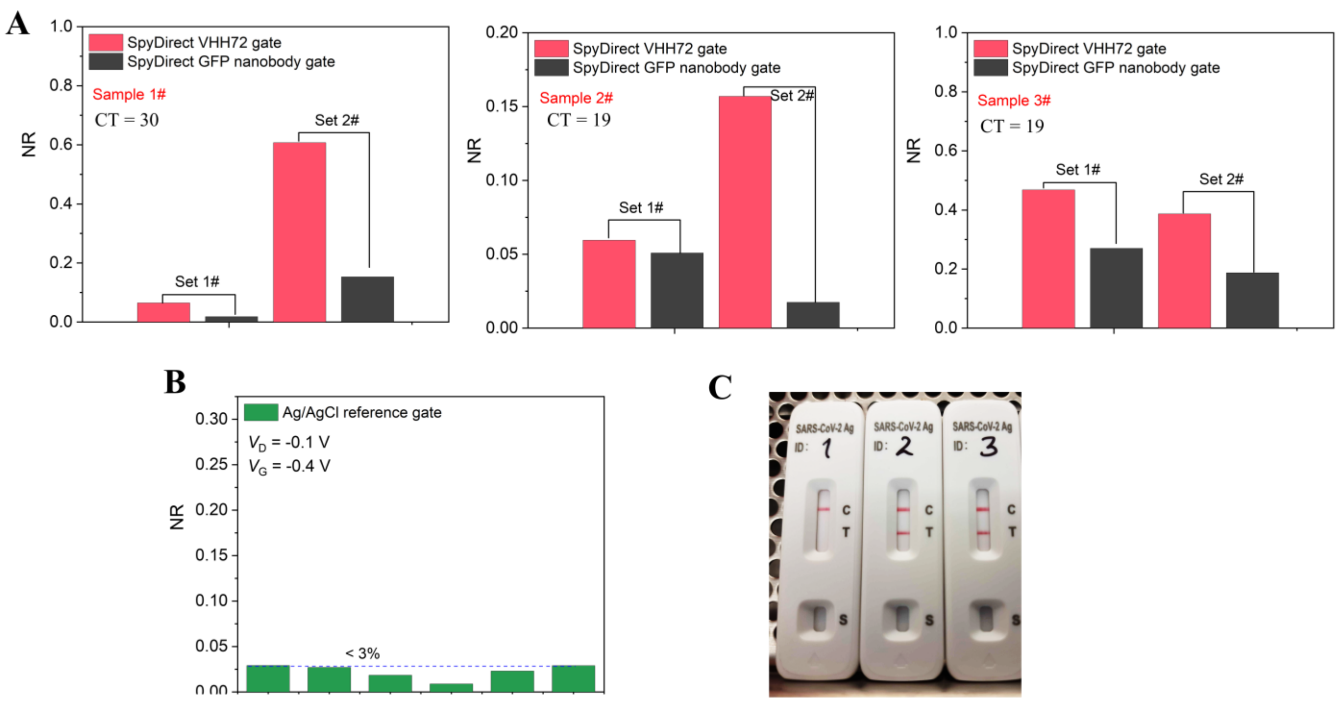

Figure S10. Proof-of-concept SARS-CoV-2 clinical sample testing. (A) Normalized response (NR) for three nasopharyngeal swab samples from COVID-19-positive hospitalized patients. Each sample was measured on the same OECT channel using gates in this order: Ag/AgCl reference

gate, SpyDirect VHH72 gate, Ag/AgCl reference gate and GFP nanobody gate. Ag/AgCl reference gate was used to monitor the stability of the OECT channel while the GFP nanobody gate served as the negative control. Two sets of gates were used for each sample. Cycle threshold (CT) values from RT-PCR are indicated. **(B)** The stability of the OECT channel was monitored using an Ag/AgCl reference gate. **(C)** Commercial SARS-CoV-2 antigen rapid tests were performed on the same samples.

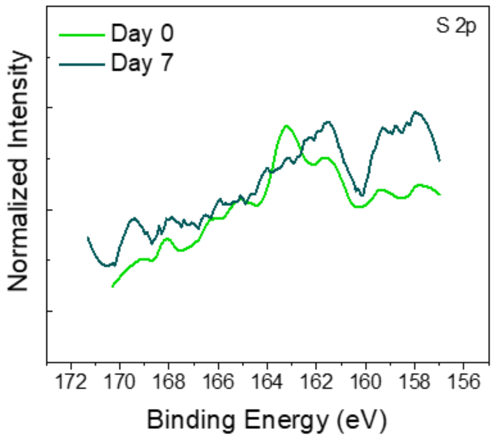

Figure S11. XPS spectra of S 1s of HDT SAM-based gates before and after 7-day storage in 10 mM PBS, pH 7.4 at 4 °C.

Table S1. RT-PCR result of COVID-19 clinical samples detail.

Sample ID	COVID-19 Result	Detected Gene	
		Gene Name	CT
1#	positive	E & N Gene	E:31 N:30
2#	positive	N2-Gene	N: 19
3#	positive	E & N Gene	E:17 N:19

References

1. M. H. Kubala, O. Kovtun, K. Alexandrov, B. M. Collins, Structural and thermodynamic analysis of the GFP: GFP-nanobody complex. *Protein Science* **19**, 2389-2401 (2010).
2. J. Ries, C. Kaplan, E. Platonova, H. Eghlidi, H. Ewers, A simple, versatile method for GFP-based super-resolution microscopy via nanobodies. *Nature methods* **9**, 582-584 (2012).
3. J. Jenzano, S. Hogan, R. Lundblad, Factors influencing measurement of human salivary lysozyme in lysoplate and turbidimetric assays. *J. Clin. Microbiol.* **24**, 963-967 (1986).
